# The walnut shell network: 3D visualisation of symplastic and apoplastic transport routes in sclerenchyma tissue

**DOI:** 10.1007/s00425-022-03960-w

**Published:** 2022-07-26

**Authors:** Sebastian J. Antreich, Jessica C. Huss, Nannan Xiao, Adya Singh, Notburga Gierlinger

**Affiliations:** Institute of Biophysics, Department of Nanobiotechnology, University of Natural Resources and Life Sciences, 1190 Vienna, Austria

**Keywords:** Serial block face-SEM, Seed coats, Plasmodesmata, Pit channels, Intercellular space, Lignification

## Abstract

**Main conclusion:**

High symplastic connectivity via pits was linked to the lignification of the developing walnut shell. With maturation, this network lessened, whereas apoplastic intercellular space remained and became relevant for shell drying.

**Abstract:**

The shell of the walnut (*Juglans regia*) sclerifies within several weeks. This fast secondary cell wall thickening and lignification of the shell tissue might need metabolites from the supporting husk tissue. To reveal the transport capacity of the walnut shell tissue and its connection to the husk, we visualised the symplastic and apoplastic transport routes during shell development by serial block face-SEM and 3D reconstruction. We found an extensive network of pit channels connecting the cells within the shell tissue, but even more towards the husk tissue. Each pit channel ended in a pit field, which was occupied by multiple plasmodesmata passing through the middle lamella. During shell development, secondary cell wall formation progressed towards the interior of the cell, leaving active pit channels open. In contrast, pit channels, which had no plasmodesmata connection to a neighbouring cell, got filled by cellulose layers from the inner cell wall lamellae. A comparison with other nut species showed that an extended network during sclerification seemed to be linked to high cell wall lignification and that the connectivity between cells got reduced with maturation. In contrast, intercellular spaces between cells remained unchanged during the entire sclerification process, allowing air and water to flow through the walnut shell tissue when mature. The connectivity between inner tissue and environment was essential during shell drying in the last month of nut development to avoid mould formation. The findings highlight how connectivity and transport work in developing walnut shell tissue and how finally in the mature state these structures influence shell mechanics, permeability, conservation and germination.

**Supplementary Information:**

The online version contains supplementary material available at 10.1007/s00425-022-03960-w.

## Introduction

In the last few years, research on the walnut shell has provided fascinating information about shell anatomy and structure, which influence the shell’s mechanical performance. The shell consists of polylobate cells, forming a 3D puzzle, with thick and strongly lignified cell walls (Antreich et al. 2019). The shape is defined during morphogenesis prior to sclerification due to cellulosic cell wall thickenings that restrict the expansion of the cell wall on different loci (Antreich et al. 2021). With the onset of sclerification, a secondary cell wall is deposited in helicoidally arranged lamellae and lignin is incorporated during maturation (Xiao et al. 2021, 2020). These processes and the 3D puzzle shape give the shell tissue greater strength and stiffness compared to other nut shells with non-lobed cell shapes (Huss et al. 2020).

Sclerification of shell tissue is a necessity to protect the developing seed in many nuts or drupes (Huss and Gierlinger 2021) and often occurs within several days (Dardick and Callahan 2014). During sclerification of the peach endocarp, for example, genes involved in lignin biosynthesis are highly upregulated and enzymes related to cell wall biosynthesis and secondary wall formation are more active (Dardick et al. 2010). Within ten days the whole endocarp of peach is sclerified, leading to an exponential increase in lignin content (Hu et al. 2011). It ranges from 20 to 48% in eudicot nut shells (Landucci et al. 2020; Li et al. 2018) and in *J. regia* from 30 to 50%, depending on cultivar (Zhao et al. 2019). During the main lignification period of walnut shells (15 days), Wu et al. (2021) revealed an upregulation of genes responsible for the transduction of plant hormone signal, the biosynthesis of phenylpropanoids and the metabolism of starch and sucrose. This suggests that the enormous increase in secondary cell wall formation and lignin biosynthesis needs to be supported by metabolites from active cells to be accomplished in this rather short time period. To distribute these components from the surrounding vascular tissue to and within the shell, an effective transport system is needed.

Symplastic pathways in living cells are linked via plasmodesmata (PD), developed from transforming endoplasmatic reticulum traversing the common cell wall. PD can be formed during cell division (primary) or during cell expansion (secondary) (Roberts and Oparka 2003; Ehlers and Kollmann 2001). Their diameters (size exclusion limit), density in the wall and structures (simple or branched) influence the transport capacity of the symplast (Faulkner 2018). Therefore, high metabolic fluxes in transport tissues lead to dense PD connections between cells (Yan et al. 2019; Danila et al. 2016; Kuo et al. 1974). PDs can be clustered in pit fields, where no cellulose is deposited during cell wall thickening and sclerification. This leads to the presence of rather long channels particularly in greatly thickened cell walls, visible in sections and cracked surfaces of various nut shell tissues (Xiao et al. 2021; Antreich et al. 2019; Flores-Johnson et al. 2018; Schüler et al. 2014; Hammami et al. 2013; Kaniewski 1965). To prevent the deposition of cellulose material during sclerification a regulating mechanism has to exist. Several studies have shown that in *Arabidopsis* xylem cells the pit channels are kept free from cellulose due to multiple proteins affecting cortical microtubule depolymerisation (Oda and Fukuda 2013, 2012; Oda et al. 2010).

Besides the symplastic transport route via the PD, metabolites can be transported through the apoplast including the intercellular space (ICS). Studies on phloem unloading sites in fruits reveal symplastic and/or apoplastic pathways to sink tissues, like seeds and endocarp (Wu et al. 2004; Patrick and Offler 2001).

All these transport routes are necessary during development, but also play an important role when the shells are mature. On the one hand, pit channels and ICS are discontinuities in the tissue matrix and therefore influence mechanical properties (Gludovatz et al. 2017) and crack resistance (Schmier et al. 2020). On the other hand, they contribute to the permeability of water and air, which has consequences for seed dormancy and germination (Werker 1980).

In this paper, we reveal the symplastic and apoplastic transport pathways in the developing walnut shell by means of serial block face-SEM (SBF-SEM), SEM and TEM. We highlight the enormous symplastic transport capacity needed during sclerification in 3D and how it changes until maturity at the cellular and subcellular levels. Furthermore, we discuss the possible function of the apoplastic pathway during walnut shell drying and its impact on the kernel. To set our findings in a broader context, we compare shell development in walnut with other fruit shells and seed coats from our own research and other published studies.

## Methods

### Samples

Walnuts (*Juglans regia, ‘Geisenheim 120’*), Arizona black walnuts (*Juglans major*), pecans (*Carya illinoinensis*) and pistachios (*Pistacia vera*) were harvested in 2019. Walnuts and pecans were obtained from the BOKU horticultural garden Jedlersdorf in Vienna, Austria, black walnuts from a plant pot in front of the university (BOKU, Vienna) and pistachio from collections in Kerman, Iran.

The walnuts used in this study for SBF-SEM belong to the series analysed in Antreich et al. (2021) and correspond to weeks 12 (July) and 18 (August). Additionally, walnuts were collected in September and October, pecans and black walnuts in July and October, and pistachios in June and September. At least 5 different fruits per species and stage were analysed.

### Staining

Lignification of the shell was shown via Wiesner and Fuchsin-Chrysoidine-Astra blue (FCA) staining on whole nuts and thin sections. For the Wiesner solution, 20 mg/ml phloroglucinol (Sigma Aldrich) was first mixed with 20% ethanol and then with concentrated 12 M hydrochloric acid (v:v = 80:20). FCA solution was prepared by mixing 0.1 mg/mL of New Fuchsin (Roth), 0.143 mg/mL of Chrysoidine (Sigma Aldrich), 1.25 mg/mL of Astra blue (Roth) with acetic acid (v:v = 1:50). For the Wiesner staining, all nuts were halved with a razor blade (developing nuts) or a band saw (mature nuts) and placed in the Wiesner solution for at least 30 min. Immediately thereafter cross-sections were photographed. Thin sections of shell tissue cut by a cryo-microtome (CM3050 S, Leica) were stained for 5 min. For the FCA staining, thin sections were cut from shell tissues by cryo- (developing) and rotary (RM225, Leica) microtomes (mature) and stained for 30 min, followed by successive washing with distilled water and an ethanol series (30, 70, 30%). The images of the stained tissues were acquired using a Labophot-2 microscope (Nikon) in a bright field.

### Maceration

Multiple shell fragments of all four mature nuts were immersed in glacial acetic acid (Roth) and hydrogen peroxide (Roth) (v:v = 1:1) for 3 h at 80 °C until the fragments turned white. After washing in distilled water, the fragments were filtered from the solution and freeze dried.

#### SEM

Detailed surface scans of the outer walnut shells (October) and cracked shell pieces (July, October) as well as single macerated cells of all four shells were performed without prior coating in an Apreo SEM (FEI) in Optiplan mode (T1, 1 kV, 50pA). For shell surface scans, the husk was removed and the shell was air-dried for several hours. To obtain cracked pieces, the dry shells were smashed with a hammer.

### Serial block face-SEM (SBF-SEM)

SBF scans were performed on walnut samples from week 12 (developing) and week 18 (mature) and on the other three developing nut species. For each species and stage, two samples were investigated. Shell pieces of the different nuts were trimmed into cubes (around 1 × 1x1mm) and prepared according to Antreich et al. (2021) with minor changes. Due to the dense shell tissue and the resulting longer diffusion of the fixation solution, the samples were fixed with 3% glutaraldehyde overnight (Agar Scientific) and post-fixed with 2% osmium tetroxide and 0.2% ruthenium red for 4 h at room temperature. After washing, the samples were immersed in 1% thiocarbohydrazide solution and post-fixed again with 2% osmium tetroxide, followed by 0.5% uranyl acetate overnight and Waltron’s lead aspartate for 30 min (65 °C). All steps were followed by several rounds of washing with distilled water. Stained samples were dehydrated with an ethanol series followed by acetone and low viscosity resin infiltration. Polymerization was done at 65 °C for 48 h. Embedded samples were trimmed (0.5 × 0.5 × 0.5 µm) and mounted on an inbuilt microtome in the Apreo SEM. Blocks were cut in 100 nm intervals and each exposed surface was scanned with the electron beam resulting in image stacks of the shell tissue. For the developing and mature walnut, four stacks each were produced, whereas, for the other nut species, two stacks were made.

#### TEM

For TEM, 80 nm thin sections were cut from the same blocks that had been used for SBF-SEM using an Ultracut UC7 microtome (Leica). Sections were placed on 300 mesh grids, post-stained with potassium permanganate, and analysed with a Tecnai T20 TEM (FEI).

### Microcomputed tomography (µCT)

µCT scans were performed in an X-ray micro-computed tomograph (EasyTom 150/160 system, RX solutions) with a Hamamatsu nanofocus tube (tungsten filament, lower limit of 0.8 µm voxel size) and a flat panel detector. Walnut shell fragments from three developing as well as mature nuts were scanned with 60 kV and 200µA and reconstructed via the software XAct2 (RX solutions) resulting in image stacks of the shell fragments.

### 3D visualisation and calculation

Image stacks of µCT and SBF-SEM were aligned and trimmed with the software ImageJ (NIH). The processed stacks were used in the Amira software (FEI) for 3D visualisation. Individual cells were segmented manually followed by semi-automatic segmentation of cell wall, lumen and ICS.

For pit area analysis in developing and mature tissue, surface areas of ICS, pit channels and cell walls to neighbouring cells were calculated for all segmented cells. Pit channel density was determined by dividing the number of pit channels by the total cell surface area. Mean single pit area was calculated by dividing the pit channel area per cell with the number of segmented pit channels. Additionally, cell wall thickness was determined using the thickness function plugin in ImageJ (Dougherty and Kunzelmann 2007). To measure the relative abundance of ICS in the developing and mature walnut tissues, six subvolumes with 50 µm^3^ were segmented into ICS, lumen and cell wall and each volume was calculated.

### Statistics

For data analysis, the software STATISTICA 7.1 (StatSoft.) was used. To compare all data from the two walnut developmental stages, Mann–Whitney *U* tests were performed as data showed no homogeneity of variances. For the pit channel densities of the different nut species, a Kruskal–Wallis one-way ANOVA on ranks was carried out followed by a multiple comparisons of mean group ranks. Results with significant differences are labelled with asterisks (* = *p* < 0.05, ** = *p* < 0.01, *** = *p* < 0.001).

## Results

**Fig. 1:**
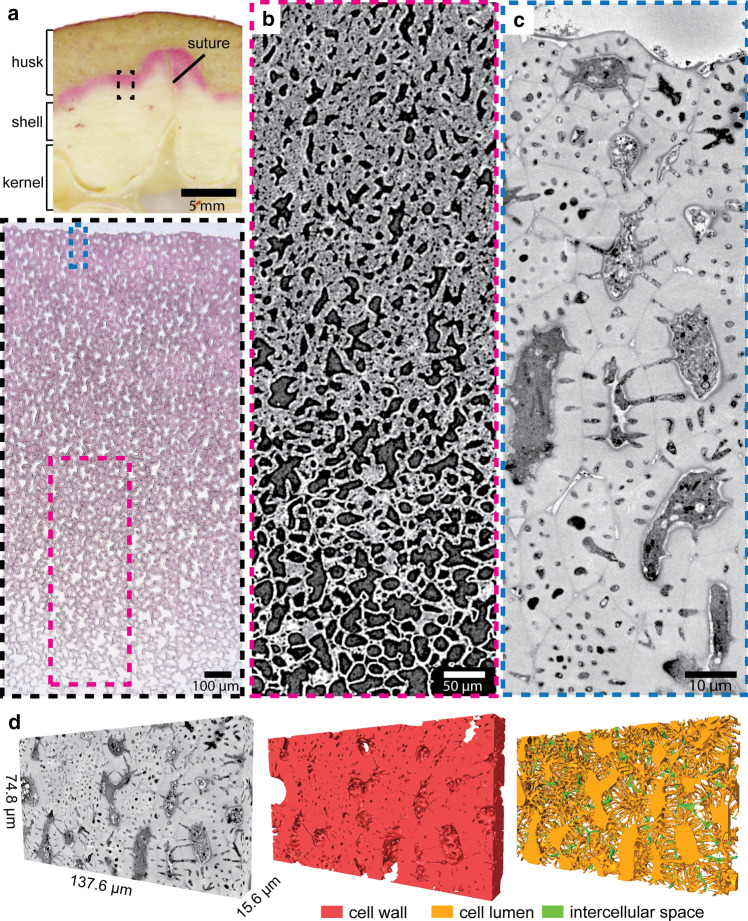
Walnut shell tissue during development: **a**) Cross-section of a developing walnut fruit stained with phloroglucinol to detect lignin aldehydes. The walnut is composed of a green husk, followed by the shell forming tissue, and the kernel. The two halves of the shell tissue are separated by the suture. The zoom-in shows a thin section of the shell tissue with the regions of interest for µCT (pink) and SBF-SEM (blue). The red staining highlights the lignification of the thick-walled cells. **b**) µCT scan of the developing shell tissue shows a gradient in the cell wall thickness towards the inside. Despite the lower resolution, pit channels and ICS are visible. **c**) Block face image of the walnut shell tissue reveals the thick cell walls of the shell tissue with numerous channels in high resolution. **d**) 3D representation of a block of the shell tissue and its 3D reconstruction. The red reconstruction shows the cell wall, orange is the cell lumen with pit channels and green is the ICS. Numerous pit channels connect the lumen of neighbouring cells forming a dense network

During walnut fruit development, a green husk covers the shell and inwards the soft shell tissue fills up completely the space between husk and kernel (Fig. 1a). Sclerified shell tissue forms first along the suture from top to bottom and then continues along the shell-husk interface all over the fruit (Antreich et al. 2021). Twelve weeks after catkin formation, the shell tissue reveals a gradient of sclerification from outside to inside, shown by µCT scans (Fig. 1b). Block face electron images of a representative area of the shell-husk interface show irregular cells with thickening cell walls and numerous small pit channels (Fig. 1c). The shell tissue is closely associated with the vascular bundles in the husk, which are leading to grooves on the mature shell. By using the SBF-SEM technique and 3D reconstruction on a thin block of this tissue, we elucidated how numerous pit channels pass through the developing cell wall (Fig. 1d, for animation see Online Resource 1). These multiple connections between neighbouring cells form an extensive transport network in the developing shell.

**Fig. 2:**
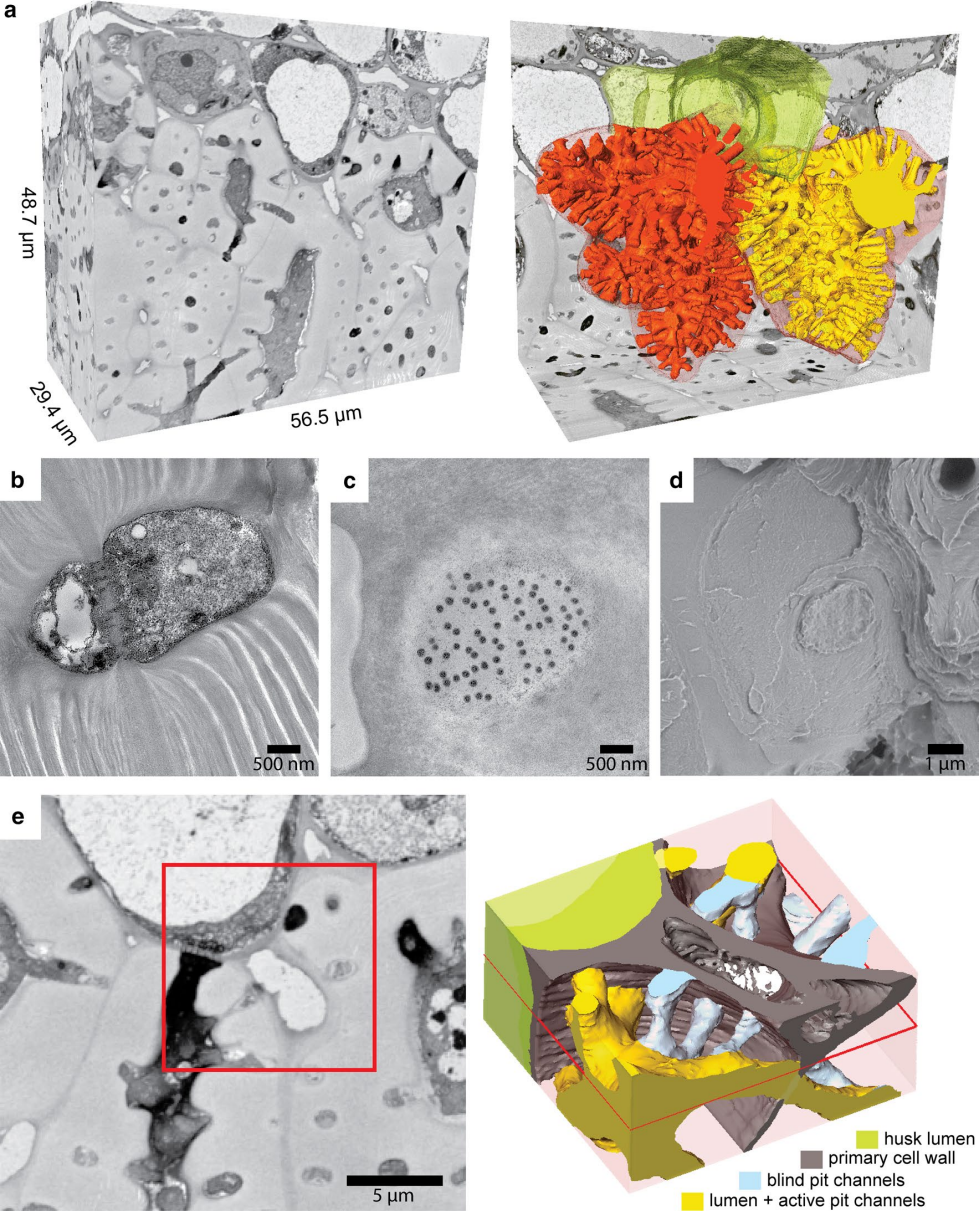
Network of pit channels through the cell walls at the interface from the shell to the husk tissue: **a**) SBF-SEM stacks of the outermost cells of the shell followed by 3D reconstruction revealed that pit channels not only connect neighbouring cells in the shell (red and yellow) but also cells of the husk (green). **b**) TEM images of connected pit channels show a high number of PD crossing the primary membrane. **c**) Cross-section through the primary cell wall reveal a high number of PD only within the pit channel. **d**) SEM image of the young shell showing a remaining pit field with primary cell wall after fracture. The image also shows the smooth surface of the ICS (left) and a fragment of the secondary cell wall (right). e) SBF-SEM images and the 3D reconstruction at the husk-shell interface at an ICS reveal connecting pit channels between cells (shell-shell as well as shell-husk, yellow) and pit channels ending blind to the ICS (blue). These channels have no PD

Detailed SBF scans and its 3D reconstruction revealed that these channels were also formed towards the interface between the husk and shell tissue (Fig. 2a. for animation see Online Resource 2). In the segmented cells next to the husk, pit channel density was as high as between the cells of the shell (Table 1). The pit channels crossed the secondary cell wall and ended at the primary cell wall (Fig. 2b). Opposite channels were connected through multiple PD in the primary cell wall (Fig. 2b, c). They were high in number and randomly distributed within the secondary cell wall-free space. The secondary cell wall encircled the pit area from the first layer on (Fig. 2d). Scans also revealed that some of the channels were not connected by an opposite channel. Pit channels that end blindly, because of an ICS or due to a shift of the opposite channel, had no PD (Fig. 2e, for animation see Online Resource 3). So pit channels not only link the surrounding husk with the shell tissue but also the apoplastic and symplastic pathways by ending in the ICS.

**Table 1:** Pit channel characteristics in different cell interfaces in developing and mature walnut shell tissue as well as wall thickness of the analysed cells

Stage of development	*n*	Interfaces	pit area per cell area (%)	pit channel density (µm^−2^)	single pit area (µm^2^)	cell wall thickness (µm)
Developing cells	10	Overall	5 (± 1)	0.07 (± 0.01)	0.8 (± 0.1)	6.0 (± 0.6)
		Cell–cell	6 (± 1)	0.07 (± 0.01)	1.0 (± 0.2)	
		Cell–husk	12 (± 2)	0.09 (± 0.02)	1.4 (± 0.3)	
		Cell–ICS	2 (± 1)	0.06 (± 0.02)	0.2 (± 0.1)	
Mature cells	10	Overall	3.3 (± 0.8) ***	0.021 (± 0.005) ***	1.6 (± 0.4) ***	9 (± 1) ***
		Cell–cell	3.4 (± 0.8)	0.024 (± 0.008)	1.5 (± 0.6)	
		Cell–husk	7 (± 2)	0.031 (± 0.007)	2.4 (± 0.5)	
		Cell–ICS	0.09 (± 0.05)	0.007 (± 0.004)	0.14 (± 0.08)	

Significant differences (Mann–Whitney *U* Test) between developing and mature cells (Overall) are indicated by asterisks. Data are shown as mean (± SD)

**Fig. 3:**
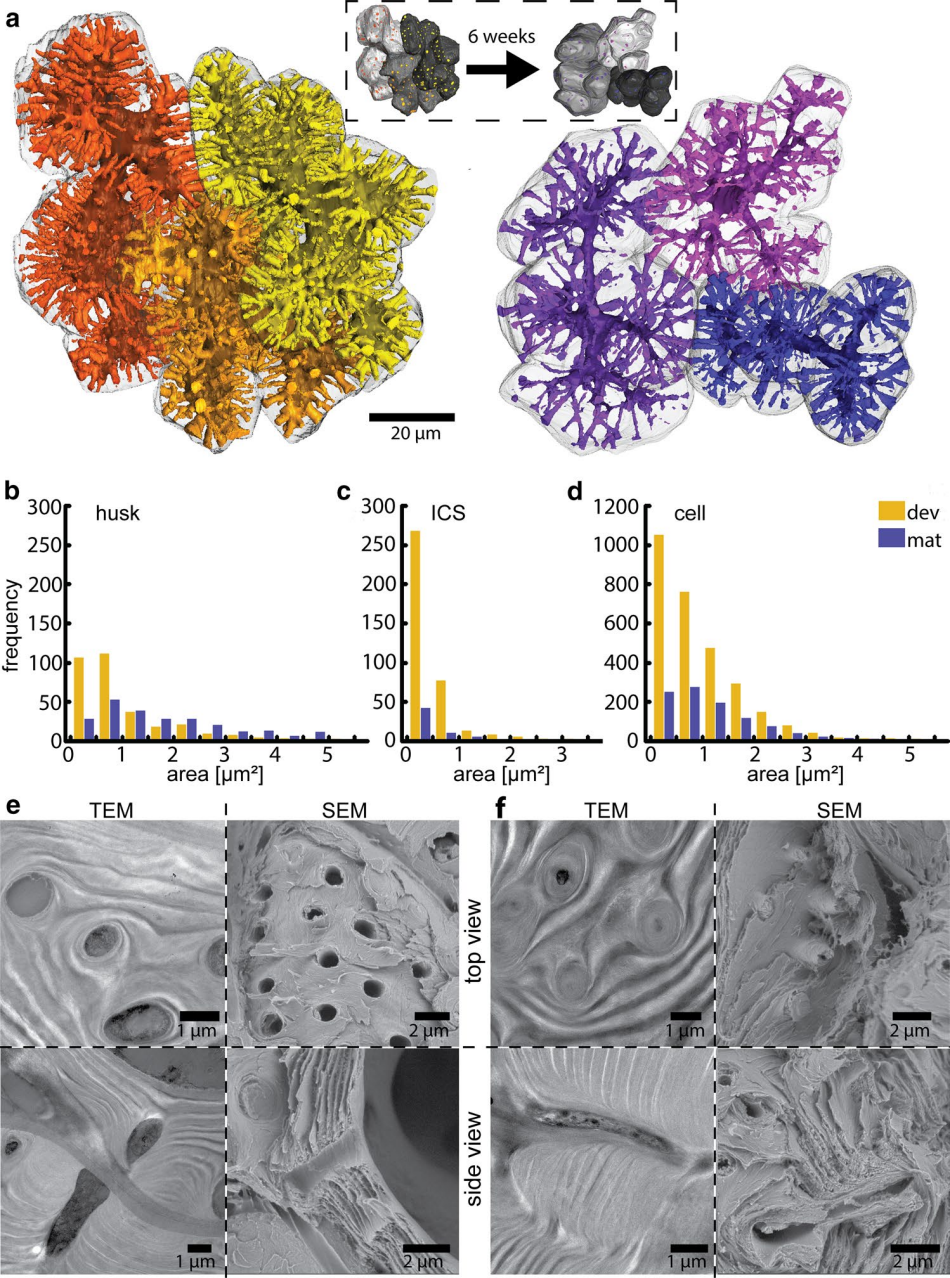
Reduced pit channel density during sclerification of the shell tissue: **a**) 3D reconstruction of the lumen and pit channels of three cells of the developing and the mature tissue six weeks later. The insert shows the shape and position of the three cells. The number of pit channels connecting the cells decreases visibly and the lumina shrink to a tube-like shape. **b**–**d**) Pit channel contact area of developing (orange) and mature (purple) cells at the interfaces **b**) cell-husk, **c**) cell-ICS and **d**) cell–cell. In the cell-husk interface mainly pit areas below 1 µm^2^ are decreasing. In the cell-ICS interface the already small pit areas become even less and smaller. In the cell–cell interface small pit channels decrease the most. **e**) TEM images show pit channels embedded into the cellulose matrix of the cell wall and SEM images of delaminated cell surfaces show round and open pit channels. Representative side views of pit channels in TEM and SEM highlight the multiple lamellae of the secondary cell wall and their slight curvature at the pit edge. **f**) SBF-SEM image of the mature shell show less pit channels and a substantial reduction in the lumina of the cells. Cross-sections in TEM and SEM display that cell wall material is closing the pit channels by growing into them. In the side view in TEM and SEM images, it is visible that the pit channel became ingrown by inner lamellae

To highlight channel dynamics, not only the developing walnut shell tissue but also a more mature stage was investigated. The 3D reconstruction of shell tissue showed that after six weeks the lumen shrank to a tube-like shape and also the channels appeared longer but fewer (Fig. 3a, for animation see Online Resource 4). Detailed analysis of individual cells of the 3D reconstruction showed an overall drop in channel density from 0.07 (± 0.01) to 0.021 (± 0.005) µm^−2^ (Table 1). In contrast, the mean single pit area increased from 0.8 µm^2^ (± 0.1) to 1.6 µm^2^ (± 0.4) but showed strong differences among the different interfaces (Fig. 3b–d). In the developing cells, pit channel density was relatively constant at all interfaces. Still, the relative pit area as well as the mean single pit area were clearly lower at the cell-ICS interface, which accounted for 15% (± 3) of the total cell surface. At the cell-husk interface, the relative pit area as well as mean pit areas were much higher than at the cell–cell interface. In the mature state, the mean pit area at the cell-ICS interface, which accounted here for 20% (± 5) of the total cell surface, almost vanished due to lower pit channel density and smaller mean single pit area. This is also seen in the distribution of the size classes (Fig. 3c). On the other hand, the mean single pit area of the cell-husk interface was much higher than the cell–cell interface. As pit channel density was also higher, relative pit area doubled at the husk interface. A closer look at the size classes showed that mainly the small pit areas were disappearing, whereas, the larger pit areas remained (Fig. 3b). In the cell–cell interface all size classes got smaller, however, the small pit channels vanished most notably (Fig. 3d). During shell development the tissue got denser due to secondary cell wall formation, so that cell wall thickness increased from 6.0 µm (± 0.6) to 9 µm (± 1) (Table 1). Comparison of TEM and SEM images made from the same blocks after SBF or made after cracking shell pieces, respectively, provided additional information on the channel dynamics. In the developing tissue, channels were wide and short due to the thinner cell walls (Fig. 3e). The lamella of the secondary cell wall encircled the channels but left the space open. However, the lamellae grew slightly into the channel so that close to the pit edge the lamellae turned towards the outside. In the more mature tissue, channels became longer due to the additional layers of cell wall material deposited at the lumen-sided secondary cell wall (Fig. 3f). However, many channels also became partly or completely filled with cell wall material because inner lamellae of the secondary cell wall grew into the pit channels. The gradual filling reduced the pit channel diameter until it was completely filled. In some cases, the inner core of the pit channels remained intact after cracking (Fig. 3f, top right).

**Fig. 4:**
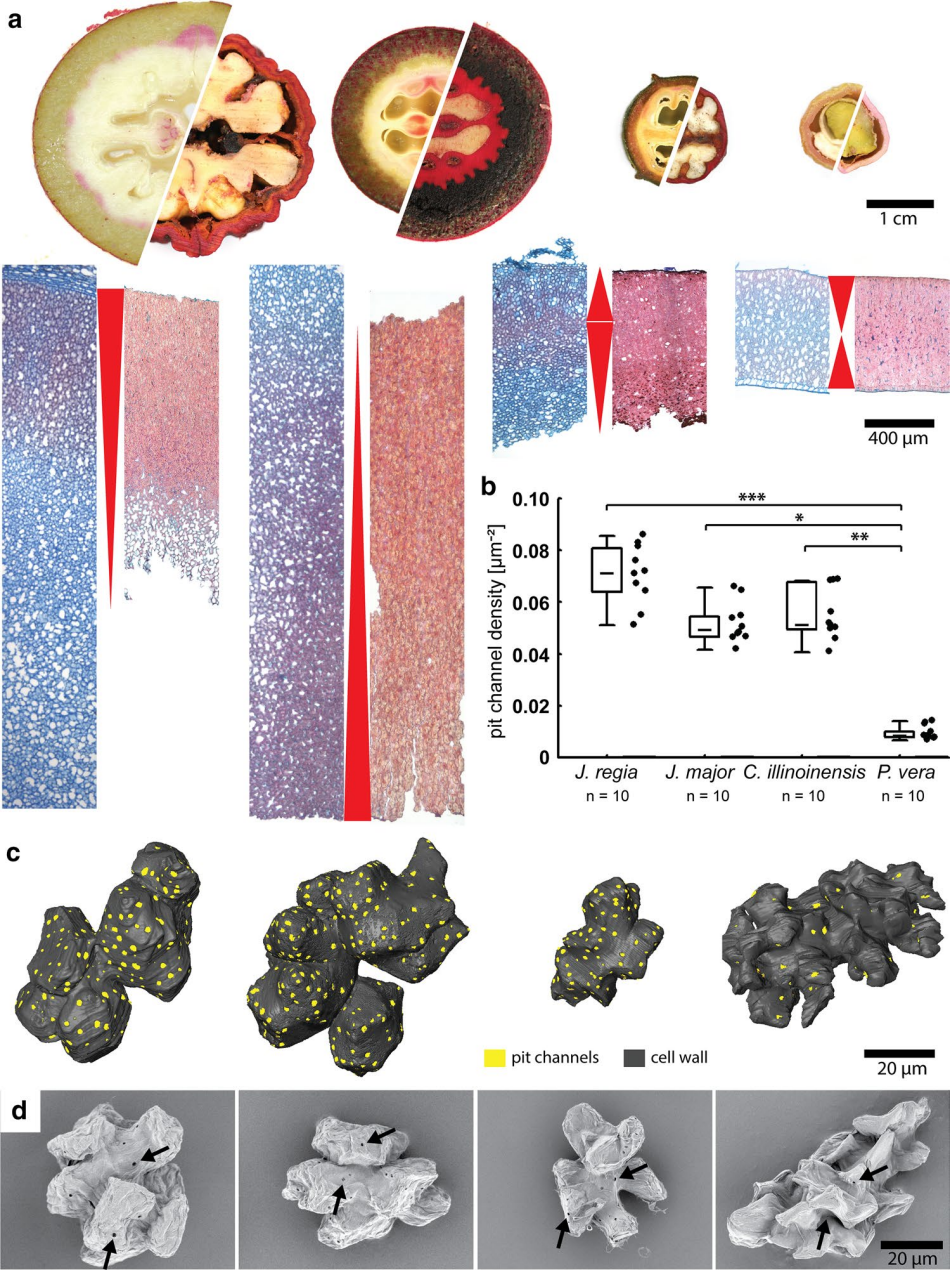
Comparison of four eudicot fruits with polylobate cells during shell development: **a**) Cross-sections show fruits in developing and mature state after staining with phloroglucinol. The strong staining of *J. regia*, *J. major* and *C. illinoinensis* indicates high lignin content after maturity compared to *P. vera*. Tissue sections show the direction of sclerification (indicated by red arrowheads) and the four different lignification patterns during shell development. The walnut shell is the only one where a lignification gradient is observable from outside to inside. **b**) Pit channel density of cells of walnut are compared with black walnut, pecan and pistachio during shell development. The closely related Juglandaceae species show a higher density than the pistachio (box: 25–75%, whisker: min–max). **c**) 3D reconstructions of representative cells of each species during development show the lobate shape of the cells and their multiple pit channels (yellow). **d**) After maceration with glacial acetic acid and hydrogen peroxide, mature cells of all species show less open pit channels (exemplary indicated by arrows) compared to the reconstructed developing cells

Shell development of four different species was studied, namely three species from the Juglandaceae family *J. regia*, *J. major* and *Carya illinoinensis* and one species from the Anacardiaceae family *Pistacia vera*. The staining on the whole nut and tissue level revealed the initial sites of lignification of developing shells and the final shape of lignified tissue of mature fruits (Fig. 4a). In *J. regia* lignin became deposited along the outermost cell layers and successively propagated towards the inside. When mature, the husk was removed and the shell covered a large cavity with the kernel. The tissue itself exhibited a gradient in sclerified cells towards the inside of the shell. The opposite could be seen in *J. major* as sclerification started at the inside and continued towards the outside. In mature nuts, the husk remained on the shell and the whole shell tissue from kernel to husk was sclerified. In the developing shell of *C. illinoinensis,* sclerification started mainly in the outer half of the shell. However, the outermost cell layers, which were shaped more wavier than lobed and often contained crystals, sclerified later. In contrast to *J. regia*, sclerification continued towards the inside up to the kernel, as seen in mature nuts and tissues. A completely different lignification pattern was found in *P. vera*. Here, lignification started both on the outside and inside and continued to the center of the tissue. Generally, *P. vera* is known to have a rather low lignin content (Landucci et al. 2020), which was also indicated by lower staining intensity. Three species were known to have polylobate cells in the shell tissue (Huss et al. 2020). Here, we showed for the first time the polylobate shape of the cells of *J. major*. 3D reconstructions of individual cells allowed for a detailed analysis of pit channels all over the irregular cell walls (Fig. 4b, c). We found that all four species differed in pit channel density during development and that the three closely related species *J. regia*, *J. major* and *C. illinoinensis* showed high densities during early sclerification, whereas *P. vera* had around 6 times less pit channels per µm^2^. However, channel density reduction, which was measured in the reconstructed cells in *J. regia*, was also observable in macerated mature cells of the other three nut species (Fig. 4d).

**Fig. 5:**
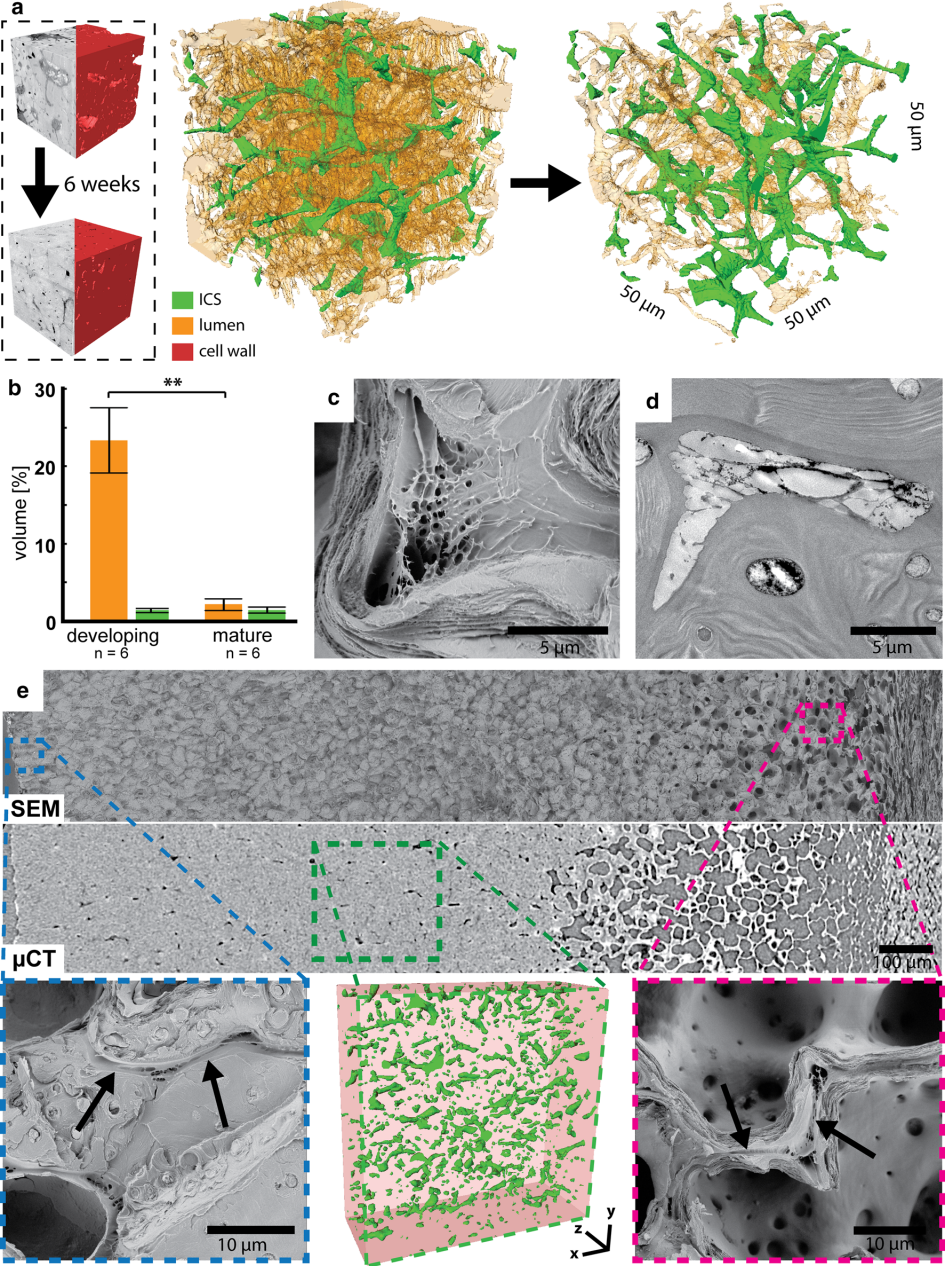
Intercellular spaces remain open during maturation: **a**) 3D reconstructions of the ICS (green) show a net-like appearance throughout the tissue in developing and mature tissues six weeks later. The small blocks show the corresponding SBF-scan and the reconstructed cell wall (red). The cell lumen is visualized in orange. **b**) The comparison of open spaces in the blocks shows a reduction of the cell lumina but not of the ICS (mean ± SD). **c**) SEM image reveals that strands were arranged in a foam-like pattern in the ICS. **d**) TEM image of an ICS with dark stained strands within the free space. **e**) Fracture faces in the SEM and µCT scans of the whole shell tissue reveal that the ICS (arrows) can be found from the outermost (blue insert) to the innermost cells (pink insert) and also throughout the shell tissue (green, 3D reconstruction from µCT)

Besides the pit channels, a second network was present throughout the shell tissue: the ICS. 3D reconstructions showed that it was visible all across the shell (Fig. 5a) and occupied around 1.3% (± 0.3) of the developing tissue volume and 1.4% (± 0.4) of the mature tissue, whereas cell lumen decreased from 25% (± 4) to 4% (± 1) (Fig. 5b). Due to the irregular cell shape also the ICS was irregular with thinner and thicker channels and several crossings as shown in Online Resource 2. ICS at cell lobe indents showed often a foam-like structure (Fig. 5c), which was darkly stained in TEM-images (Fig. 5d). The ICS was found all over the shell tissue creating a continuous channel network between the cells (Fig. 5e). It even reached the surface of the shell connecting the ICS with the husk ICS. They remained open after maturation giving them the appearance of pores when seen from the outside.

**Fig. 6:**
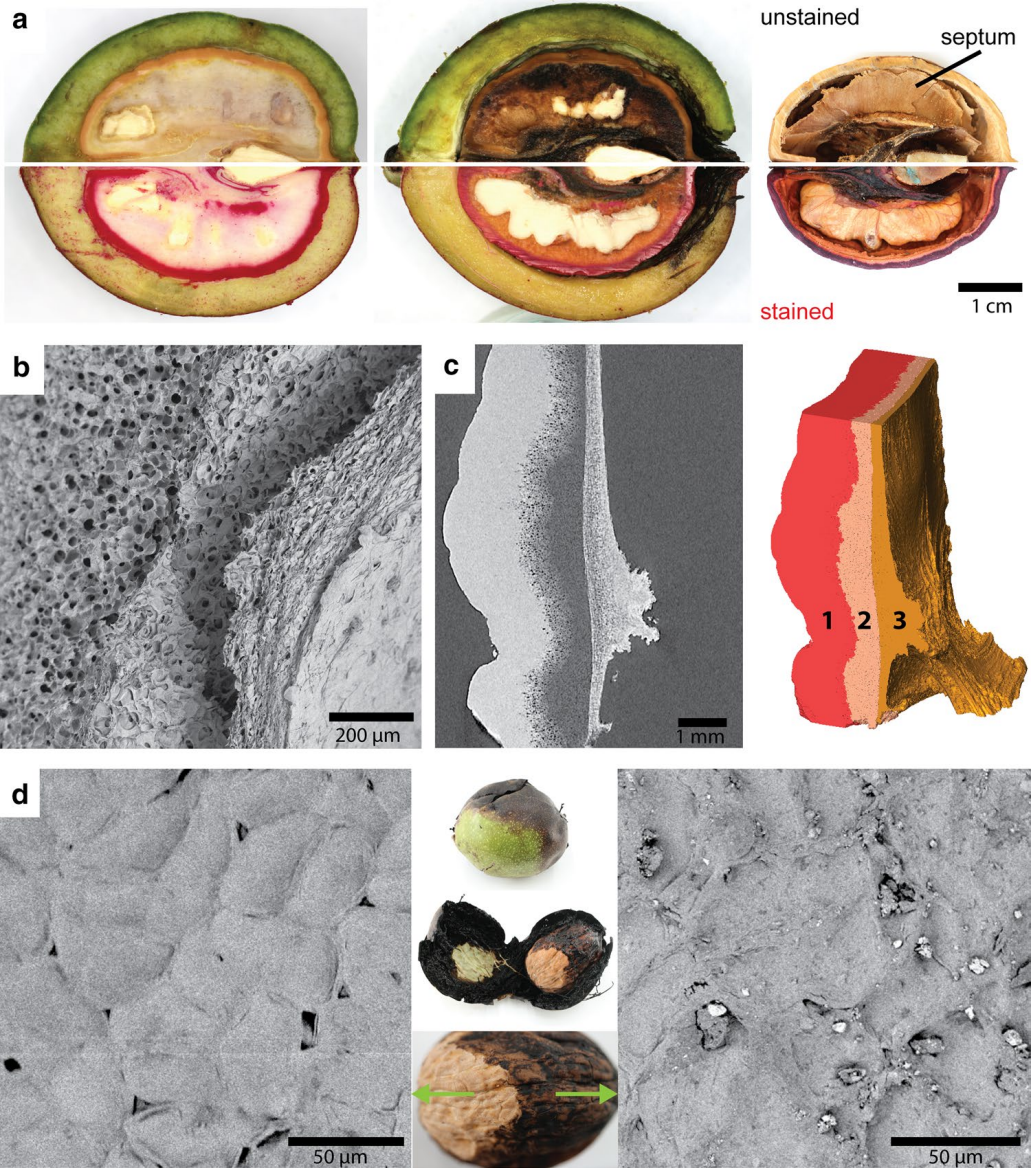
ICS is essential for tissue drying in the final phase of fruit development: **a**) Photographs of unstained and phloroglucinol-stained walnuts in August, September and October show a constant drying of the inner unlignified tissue which causes the formation of the cavity within the mature nut. This process starts first at the top but later occurs all around the kernel. **b**) SEM image of the shell shows the collapsed inner tissue separated from the outer shell tissue. **c**) A close-up µCT-scan and 3D reconstruction show the difference between the sclerified dense (1) and porous outer shell (2) and the collapsed inner shell tissue (3), which results in a dense appearance too. Part of this tissue later becomes the septum between the kernel halves visible in the mature nut (see 6a). **d**) Top view (surface) of a mature walnut shell, which is partly covered by a rotten husk (middle). SEM images reveal open pores of the ICS on the clean surface, where the husk detached from the shell naturally (left) and blocked pores on the stained surface, where the husk was rotting (right)

After sclerification of the outer walnut shell in August, the inner still soft tissue started to decay and dry until the fruit droped (Fig. 6a). During this time period, the husk detached from the shell, so that the pores of the ICS were uncovered. Finally in October, the inner non-sclerified shell tissue dried and collapsed forming a dense layer along the inner sclerified tissue (Fig. 6b). This gave the mature shell a layered structure: 1) the sclerified and dense layer makes up most of the shell and was followed by 2) a partly sclerified and porous layer and 3) the non-sclerified but collapsed inner layer (Fig. 6c). The septum was formed from the collapsed tissue between the kernel halves.

During this drying phase, the detachment of the husk from the shell was often disturbed by infection with larvae, probably, of the walnut fruit fly (*Rhagoletis completa*). The larvea fed on the husk causing parts of the tissue to rot and stick on the shell (Fig. 6d). Compared to uninfected husk, the shell became stained and the pores of the ICS partly blocked by rotten husk material.

## Discussion

### High symplastic connectivity during cell wall lignification

Sclereids with multiple pit channels can be found in shell tissues of different species (Sebaa and Harche 2014; Hammami et al. 2013; Kaniewski 1965). Also our previous studies of the walnut shell report a very dense channel network between neighbouring cells (Huss et al. 2021; Xiao et al. 2021, 2020; Antreich et al. 2019). With a detailed SFB-SEM study, we reconstructed for the first time the cell connections in 3D during the sclerification process in the shell. The symplastic connectivity was present between all the neighbouring cells in the shell but also extended to the husk tissue. Analysed pit channels in the developing shell were simple tubes with a mean size of about 1 µm in diameter but showed high variability depending on the interfaces. At the ICS interface, pit fields were in the size range found in maize and *Arabidopsis* with around 150 nm (Fujita and Wasteneys 2014; Mueller and Brown 1982) or in bamboo ranging from 100 to 300 nm (Chen et al. 2020). Pit fields at the husk interface were more similar to pitted metaxylem cells with a pit diameter of about 2 µm (Fujita and Wasteneys 2014).

In walnuts, single pit channels gave space to multiple PD, which were sometimes arranged in pairs and along a line (Fig. 2c). Faulkner et al. (2008) studied pit fields in tobacco leaves at the interface between epidermis and trichome basal cells. They found pit fields with a clustered arrangement of paired PD, which, according to them, are secondary PD formed via twinning of existing PD. Densities of PD in pit fields were not determined quantitatively but could be estimated to be around 90 PD per µm^2^ pit area, if Fig. 2c is taken as an average pit field.

Besides PD density in pit fields, pit field number and area is also of great interest when it comes to symplastic transport capacity. Danila et al. (2016) compared PD density and pit field area to cell interface area between mesophyll and bundle sheath in leaves of *Setaria viridis*, maize, rice and wheat. They found relative pit field areas between 2.8 and 12.7% in mesophyll-bundle sheath interfaces and between 3.7 and 14.4% in mesophyll-mesophyll interfaces, respectively. Such values are similar to our findings in relative pit field areas in shell-shell interfaces (6% ± 1) and husk-shell interfaces (12% ± 2) in developing tissue. However, not all pit channels were connected to opposite pit channels. 2% (± 1) of the cell-ICS interface area belonged to blind pit channels and have to be excluded from symplastic transport estimations. If we consider this reduction in the pit field area and take the estimated high PD density, we would reach approximately 5 PD per µm^2^ of the cell wall. This value is slightly lower than estimated by Danila et al. (2016) in maize (7.5–11.2 PD µm-2) and in *S. viridis* (6.4–9.3 PD µm-2), but much higher than the values for *Arabidopsis* root endodermis-phloem pole pericycle interface (0.25–1.75 PD µm-2, Yan et al. 2019). Evidently, the high number of PD per cell interface in walnut is comparable to other transport tissues and seems necessary for an increase in the symplastic transport capacity of metabolites needed for sclerification of the cells from the husk to the shell and further into the shell tissue.

In general, sclerified tissues with a high lignin content appear to have a high pit channel density. Pit channel density in highly lignified *J. major* and *C. illinoinensis* tissues (each 42%, Landucci et al. 2020) was similar to that in walnuts, whereas in the less lignified pistachio (17%, Landucci et al. 2020), the density was much lower (Fig. 4b). In contrast to the highly lignified pine or *Acrocomia* fruit shells (each around 40%, Queirós et al. 2020; Rencoret et al. 2018), both of which have many thin but randomly distributed pit channels (Antreich et al. 2019; Flores-Johnson et al. 2018), we found relatively thick pit channels in walnut. However, biophysical models predict a significant reduction in effective symplastic permeability when PD are clustered into pit fields compared to a random distribution (Deinum et al. 2019). Furthermore, thin pit channels present in large numbers increase the contact surface between cytoplasm and cell wall which could be beneficial for faster incorporation of lignin as the maximal diffusion distance decreases (Xiao et al. 2021).

### Reduction of pit channel density to strengthen the mature shell tissue

Pit channels and pit fields have also an influence on the mechanical properties of cells. As shown in thick-walled Norway spruce fibres, bordered pits or pit fields enhance crack initiation during tensile loading (Eder et al. 2008). Compared to bamboo fibres with a very low pit channel density and small channel diameter, pit channels in bamboo contribute rarely to fracture events (Chen et al. 2020). Therefore, more pit channels lead to more discontinuities in the secondary cell wall, which would probably weaken the cell tissue by facilitating crack formation. Hence, the clustering of pits in the walnut may be a compromise between high symplastic transport capacity and mechanical strength. Anyway, during sclerification the walnut pit channels without cell–cell connection are filled up with secondary cell wall material to increase its mechanical strength.

The loss of adjacent pit channels occurred mainly close to cell corners or ICS, where the cell walls were separated (Fig. 2e). Roberts et al. (2001) showed in the developing mesophyll tissue in tobacco leaves that the frequency of simple PD gets reduced due to the formation of ICS during enlargement and separation of the mesophyll cells. Similarly, during morphogenesis of the walnut shell tissue the cells undergo a substantial transformation from isodiametric to polylobate shape, which leads to the formation of a more curved and branched ICS (Antreich et al. 2021). More pit channels may be connected during the early phase of morphogenesis but separation occurs after the cells change their shapes.

The observed closure of non-connected pit channels during sclerification could arise from the disappearance of proteins regulating cellulose deposition, as shown in *Arabidopsis* (Oda et al. 2013). Here, a complex of three proteins (MIDD1; Kinesin-13A and ROP11) located at the plasma membrane enhances the disassembly of cortical microtubules needed for cellulose deposition. Activated ROP11 is located in the pit plasma membrane associated with MIDD1, which binds to cortical microtubules growing towards the pit region. The further association of Kinesin-13A leads to the depolymerisation of these microtubules. In pit channels of the walnut cells without PD, we speculate that this or a similar process stops and makes it possible for the cortical microtubules to align in the pit channel over time and allow the deposition of cellulose along the sides of the channel. This leads to the ring-like filling and finally blocking of several pit channels during the ongoing sclerification, as observed in TEM cross-sections (Fig. 3f). Reis et al. (2004) showed similar fillings into pit channels in cherry pericarp and Sebaa and Harche (2014) in *Argania spinosa*. This ingrowth into the pit channels led to a Lego^©^-brick-like interlocking of the lamellae (Xiao et al. 2021) and caused the nubby appearance of cracked cells because cracks propagated along the ingrowth of the cellulose layers. Schmier et al. (2020) found similar features in cracked coconut endocarp and assumed that pits deflect cracks into the cell. Why the channels started to form at the ICS even without a PD connection, remains unknown. Maybe cells became separated in the early phase of secondary cell wall formation or the proteins regulating cell wall deposition persist for a long time and are degraded only slowly. The second statement could explain the filling of the pits mainly from the innermost later formed cellulose layers.

However, the closure of channels in the cell walls increased overall tissue density. Similar changes were shown in coconut endocarp tissue, where the mature cells had a reduced pit channel density compared to younger tissues, thereby improving macro-mechanical properties (Gludovatz et al. 2017). Also in pistachio, densification of the shell tissue during maturation has an influence on the mechanical behaviour (Xiao et al. 2021). Besides, the closure of the pit channel in the outer shell layers could affect the observed overall shell density gradient. Compared to other fruits with a husk covering the shell during development, the Persian walnut was the only species, with a big cavity between shell and kernel at maturity (Fig. 6a). As sclerification started in the outermost cells, these cells were filled with secondary cell wall and lignin first (Xiao et al. 2020). We assume that during the densification of the outer shell tissue, the number of pit channels declines and consequently reduces the transport capacity of nutrients to the inner layer. Over time, the remaining channels in the outermost cells get blocked by cell material, leading to cell death. With the lack of nutrients, the inner cells, which initiated secondary cell wall formation later, also cease to be active. This probably leads finally to the presence of gradually sclerified and non-sclerified inner tissue, missing in other species (Fig. 4a). The relative thickness of the sclerified outer tissue compared to the inner non-sclerified tissue varies with cultivars but depends also on the photosynthetic activity, as shown by Zhao et al. (2019). They found a correlation between walnut shell thickness and sun exposure, where 70% shading during development resulted in a 30% thinner sclereid layer. Similar findings were made by Li et al. (2018) on other walnut cultivars. Consequently, with less metabolites transported during the sclerfication phase the less inner cells can be sclerified until the outermost cells are sealed off.

### Special features of the ICS in the developing walnut tissue

In addition to the symplastic transport pathway, a substantial apoplastic pathway via the ICS exists in walnut shells. Prat et al. (1997) showed on parenchyma cells of *Vigna radiata* hypocotyl that the ICS of irregular polyhedral cells is already complex and connects all cells within the tissue. The ICS of the walnut shell tissue is more irregular due to the polylobate cell shapes and is formed during morphogenesis (Antreich et al. 2021). In walnut shell, the ICS took up around 1.4% of shell tissue volume regardless of tissue age and was connected to the ICS of the husk tissue. Although the ICS occupied only a small volume, the area was largely increased through the net-like form, touching around 15% of the cell surface. As the phloem unloading in the husk of walnuts occurs via the apoplastic pathway (Wu et al. 2004), this would allow an additional exchange of photoassimilates between the two tissues. Further, the apoplast pathway in phloem unloading is also associated with sink tissues, which accumulate high concentrations of photoassimilates (Patrick 1997); this also applies to the developing shell tissue.

The rather large surface of the ICS could also play a role in lignification. Lignification often begins at the cell corner through polymerisation of the free-moving lignin monomers via oxidative radicalization and involves peroxidases and/or laccases (Tobimatsu and Schuetz 2019; Wang 2013). Especially, peroxidases appear mainly in cell corners and are linked to reactive oxygen species in the apoplast (Hoffmann et al. 2020). This may also be true for walnut, where lignification begins at the cell corners and the ICS (Xiao et al. 2020), and also for the other two Juglandaceae, which also had a complex ICS system and a high lignin content in their shells. Therefore, the more cell contact with the apoplast, the greater simultaneous occurrence of the initial lignification process, which could accelerate lignification in the shell tissue. In contrast, pistachios, which lack an ICS in the shell tissue (Xiao et al. 2021), also exhibit a much lower lignin content in the shell tissue (Landucci et al. 2020).

A special feature in the ICS was the presence of foam-like protuberances. In general, pectic protuberances can be found in many different species across all vascular plants, including ferns, and can be of various shapes (Potgieter and Van Wyk 1992), and shape variability is likely to be due to the nature of cell wall separation (Paiva and Machado 2008; Carr et al. 1980). In our case, protuberances formed irregular strands in the cell corner connecting opposite cells. During morphogenesis of the polylobate cells of walnut, the pectin-rich cell wall splits at these sites due to cellulose thickenings, which enhances the formation of ICS (Antreich et al. 2021). Whether such strands are only a cause of tearing in the middle lamella or they also perform specific functions, such as forming a physical barrier against invading pathogens (El Ghaouth et al. 1994) or a biochemical barrier against oxygen (Werker 1980), remains elusive.

### The influence of the ICS on shell mechanics and permeability after maturation

In regard to mechanical aspects, again ICS constitute discontinuities in the shell tissue, which could favour crack propagation between cells. As the ICS was not filled by cell wall material like the pit channels, crack propagation in walnut may be reduced because of the high lignin content along the middle lamella (Xiao et al. 2020), counteracting cell separation. Additionally, the ICS exhibited many bends and junctions due to the complex shape, which would force cracks to be frequently deflected. This together with the high lignification and the pit structures may cause the brittle crack behaviour observed under tension (Xiao et al. 2021; Huss et al. 2020).

In regard to physiological aspects, the ICS of the shell allows water and air diffusion throughout the entire tissue. In contrast to *J. major*, where the shell tissue sclerified completely and the husk remained on the shell, *J. regia* formed a cavity around the kernel. Especially, the non-sclerified inner shell tissue will still contain residual water after sclerification of the outer shell. Drying of this tissue was rather slow and takes several weeks (Fig. 6a). It seems to occur mainly via the ICS and the uncovered pores on the shell surface after the husk detaches from the shell. So it is essential, that the husk detaches from the shell free of residues. How important this drying process is in terms of seed conservation, can be seen during pest infestation. If the outer husk is damaged by the larvae of the walnut husk fly, tannins from the husk colour the kernel and there is a greater chance of kernels being mouldy (Solar et al. 2020). We assume that, if the husk rots on the shell due to larval attack, tannins diffuse into the ICS and the husk material blocks pores, leading to incomplete drying of the kernel. Consequent presence of a rather high level of moisture in the shell can promote mould growth. Further, the ICS of the dried shell could facilitate water uptake in the spring, promoting seed germination. For example, during sample preparation infiltration of fixative solutions, including osmium, showed a deeper penetration of the tissue via the ICS in the older tissues, because the small pit channels were clogged and the diffusion through the highly lignified cell walls was restricted. So, the shell of the walnut is not sealing the kernel from the environment but rather seems to be mainly a mechanical barrier. Vahdati et al. (2012) showed that to break physiological dormancy only chilling for several weeks is needed and the presence of the shell reduces the germination rate. This is a different strategy compared to the envelope of the cork-oak (*Quercus suber*) acorn. Here, the seed is covered mainly by an external thick cuticle and a dense palisade layer without ICS, a structural design that prevents water loss (Xia et al. 2012; Sobrino-Vesperinas and Viviani 2000). Therefore, we assume that the walnut shell has to be air and water permeable for proper storage of the kernel inside the shell and to facilitate its germination in spring.

## Conclusion

Many fruits have rigid shells consisting of sclereids with multiple pit channels. Accelerated build-up of their cell walls during sclerification would no doubt require large amounts of enzymes and building blocks, for which an effective transport system is required. Highlighting the transport routes of the sclerifying walnut shell helps to understand the concept of shell making in other fruits.

However, nuts may use different structural designs and developmental strategies for optimising their performance and survival. Structures like pits and ICS, which are necessary for developmental processes such as cell shaping, will finally also influence the shell's mechanical properties, drying, permeability and germination. So, the structure–function is a continuous and dynamic relationship, during shell development, which follows the premise that every structure has its function at an appropriate time, but not all the time.

### Authors contribution statement

Conceptualization was done by SJA; data acquisition and analysis were done by SJA, JCH, NX, and AS; funding and resources were done by NG; the original draft was done by SJA; interpretation and review were done by all authors.

## Supplementary Information

Below is the link to the electronic supplementary material.

Supplementary file1 (MP4 23980 KB)

Supplementary file2 (MP4 21616 KB)

Supplementary file3 (MP4 22743 KB)

Supplementary file4 (MP4 16492 KB)

## Data Availability

The data that support the findings of this study are available from the corresponding author upon reasonable request.
